# When Is Group Membership Zero-Sum? Effects of Ethnicity, Threat, and Social Identity on Dual National Identity

**DOI:** 10.1371/journal.pone.0130539

**Published:** 2015-06-22

**Authors:** Michael Smithson, Arthur Sopeña, Michael J. Platow

**Affiliations:** 1 Research School of Psychology, The Australian National University, Canberra, A.C.T., Australia; 2 Natori City Board of Education, Natori City, Japan; University of Tuebingen Medical School, GERMANY

## Abstract

This paper presents an investigation into marginalizing racism, a form of prejudice whereby ingroup members claim that specific individuals belong to their group, but also exclude them by not granting them all of the privileges of a full ingroup member. One manifestation of this is that perceived degree of outgroup membership will covary negatively with degree of ingroup membership. That is, group membership may be treated as a zero-sum quantity (e.g., one cannot be both Australian and Iraqi). Study 1 demonstrated that judges allocate more zero-sum membership assignments and lower combined membership in their country of origin and their adopted country to high-threat migrants than low-threat migrants. Study 2 identified a subtle type of zero-sum reasoning which holds that stronger degree of membership in one’s original nationality constrains membership in a new nationality to a greater extent than stronger membership in the new nationality constrains membership in one’s original nationality. This pattern is quite general, being replicated in large samples from four nations (USA, UK, India, and China). Taken together, these studies suggest that marginalizing racism is more than a belief that people retain a “stain” from membership in their original group. Marginalizing racism also manifests itself as conditional zero-sum beliefs about multiple group memberships.

## Introduction

Marginalizing racism is a form of prejudice that, until recently, has received little attention from social psychologists. It is different from previously examined forms of prejudice as it occurs when individuals are given contradictory messages about their group membership. Ingroup members simultaneously claim that certain individuals belong to their group, but also exclude them by not granting them all of the privileges entailed by being a full ingroup member. These individuals consequently are marginalized, neither completely in nor out of the group [1].

An example of this was noted in [2], describing the status of Turkish immigrants in Germany. It suggested that there are Turks who are fluent in German, citizens by birth, and productive members of the economy. Yet “however high they rise, however good their German, Turks are not allowed to forget they are foreign” and, as one Turk expressed it, “We’re in, but not in all the way” [2]. Taken from a marginalizing racism perspective, this prejudice would be due to the position of these members within the group. On the basis of their ethnic origins, these individuals remain on the outer reaches of the group, and are never really accepted as true group members.

The social identity approach (encompassing both self-categorization theory and social identity theory) provides a solid foundation for understanding marginalizing racism, as the processes of social and self-categorization have been strongly linked to prejudice [3]. Social identity theory (SIT), in particular, has been used to explain a wide range of inter-group phenomena, including inter-group discrimination [4]. A key assertion of SIT is that two different but interrelated concepts known as the personal identity and social identity underlie one’s self concept. The personal identity reflects the characteristics and attributes that make an individual unique whilst the social identity reflects the individual’s membership in a group. Self categorization theory (SCT) built upon these notions, and is based on the related ideas that: (1) categories are used to organise people and objects [5], and (2) psychological group memberships, derived from self-categorizations, define one’s social identity. Critically, SCT allows the possibility for an individual to belong to many groups and have many social identities, although the social identity that becomes salient at any time depends on contextual factors [6]. Examples such as the Turks in Germany, however, suggest that group membership may not be a simple dichotomy (either in or out), but may be a matter of degree. In this context, group members subject to marginalizing racism may be cognitively represented by others as individuals who have partial group membership but are not considered prototypical, full members of the group. This perceptual gradation allows for the allocation of marginal or partial group membership, the very outcome of marginalizing racist processes.

SIT and SCT concepts also provided the basis for the common ingroup identity model (CIIM) [7], in which the formation of an inclusive, superordinate ingroup identity that encompasses both ingroup and outgroup members is hypothesized to reduce inter-group rivalry and prejudice. The CIIM has found support in both laboratory experiments (e.g.,[8]) and field studies (e.g.,[9]). Nonetheless, some researchers have observed the instability of the CIIM effects and raised the possibility that some members of former outgroups may not be fully accepted and can be excluded again [10].

External factors that trigger or sustain prejudice, therefore, also are worth considering. An obvious candidate is perceived threat. Perceived threat from an outgroup is a strong predictor of prejudice against that group [11]. Threat, therefore, also may trigger a process of marginalizing racism of those group members whose characteristics, attitudes or behaviours are perceived as threatening. Indeed, certain forms of threat may well be perceived as zero-sum in and of themselves. For example, intergroup threats over limited and valued resources are competitive in nature [12, 13]. Viewing group membership as zero-sum in such instances may well be an accurate representation of the material reality facing group members. Other forms of threat, however, may not have such material, zero-sum properties.

A comprehensive framework of intergroup threat, and its potential to impact upon prejudiced attitudes, has been outlined by [14] in their Integrated Threat Theory (ITT). The ITT states that prejudice is a result of four factors: negative stereotyping, intergroup anxiety, and both symbolic threat and realistic threat. Realistic threats include, but are not limited to, the well-being of ingroup members and their material resources, as we noted above. Symbolic threats, however, pertain to differences in morals, attitudes, beliefs and values that attack the foundations underpinning the way in which a group views the world and its place in it. Here, the material zero-sum nature of this threat is much less clear. If group members do see symbolic threat in zero-sum terms, however, then we anticipate that this would translate into zero-sum thinking regarding the affordance of group membership per se.

The predictions from the ITT and the CIIM regarding degree of ingroup membership are clear. The ITT predicts that, *ceteris paribus*, the degree of group membership allocated by one ingroup member to another will covary inversely with degree of perceived threat from the other. The CIIM predicts a greater degree of ingroup membership will be allocated when a superordinate group identity is invoked than when separate group identities are made salient.

The implications of the ITT and CIIM frameworks regarding *combined ingroup and outgroup membership* are, however, not as obvious. One possibility is that the degree of outgroup membership will covary negatively with degree of ingroup membership. In other words, group membership may be treated as a zero-sum quantity, whereby one cannot be both German and Turkish. Indeed, [15] produced evidence that ethnic and Muslim identification are negatively related to Dutch identification in the Netherlands. But there are obvious counter-examples where group membership is not zero-sum. Few people would insist, for instance, that one cannot be identified as German and a parent at the same time. The question of when group memberships are zero-sum seems to be a neglected topic in social psychology.

These considerations motivate an investigation of the *sum* of ingroup and outgroup membership. In Study 1, we test four tentative hypotheses derived from the ITT and CIIM regarding this sum. From the ITT, we propose that the likelihood of treating degree of membership as zero-sum will covary positively, and the sum of memberships will covary negatively, with perceived threat. From the CIIM, we predict that the sum will be greater, and the likelihood of zero-sum membership will be lower, when a superordinate group identity is invoked than when separate group identities are made salient.

Finally, in examining our hypotheses, we include ethnicity as a key factor due to the ethnic or cultural aspect in many real-world instances of marginalizing racism. We expect immigrants whose ethnic and cultural heritages are similar to natives of the country to which they immigrate will encounter less marginalizing racism than immigrants whose ethnicity differs from the natives. In Study 1, targets are described as immigrants who have obtained Australian citizenship, and Australian participants are then asked to judge the degree to which each target is “Australian” and the degree to which each is a member of his or her country of origin [16]. Canadians, who are predominantly of the same ethnic and cultural heritage as most Australians, were selected as being less likely to be marginalized than Syrians who, in turn, are selected because they have a predominantly different heritage from most Australians. Thus, we predict that Canadians will be allocated greater membership sums and will be less likely to have membership treated as zero-sum than Syrians. In summary, our hypotheses are:

1. High threat targets yield:
  - Higher likelihood of zero-sum membership assignments, and
  - Lower summed membership.
2. An inclusive superordinate in-group identity for low-threat targets yields:
  - Lower likelihood of zero-sum membership assignments, and
  - Higher summed membership.
3. Canadian targets compared to Syrian targets yield:
  - Lower likelihood of zero-sum membership assignments, and
  - Higher summed membership.

All of the hypotheses presented here are main-effects hypotheses, but we also investigate potential moderator effects. For instance, it is plausible that the impact of invoking a superordinate group identity may be reduced if threat is sufficiently high. Nevertheless, we have no strong theoretical basis for proposing specific moderator effects hypotheses.

## Study 1

### Method

#### Design

The study employed a 2 (threat level: high/low) x 2 (social identity prime: inclusive/exclusive) x 3 (marginal member ethnicity: Canadian/Syrian/no nationality) x 6 (threat topic: environment/violence/dogma/language/health/welfare) mixed design. Threat level and social identity prime were between subject variables, whereas ethnicity and threat topic were within subjects variables. Threat topic had six levels. Topic originally had seven levels (work ethic), but was reduced to six levels due to a failure to reverse the threat level for the work ethic topic in half of the questionnaires. The two dependent variables were the degree to which participants viewed the target as Australian and non-Australian (i.e., Canadian, Syrian, or non-Australian if national origin was not specified).

#### Participants

After the study was granted approval from The Australian National University (ANU) Human Research Ethics Committee (protocol 2007/2270), participants were recruited through advertisements that were placed on notice boards around the ANU campus. The advertisement asked for Australian citizens who were interested in a participating in a study examining “Attitudes about being Australian and of other Australians”. There were a total of 135 participants (93 females) with a mean age of 20 (SD = 3.72; ranging between 17 and 41). Participants were all volunteers, some of whom were or first year-psychology students who completed the study as part of their course requirement. Of the 135 participants, 105 self-identified as Anglo-Saxon, 29 identified with a different ethnicity, 118 reported English as their first language and 111 were born in Australia. Four participants were excluded from the analyses because they did not list their nationality as Australian.

#### Procedure and Materials

The study was conducted in classrooms and consisted of 1–12 people per session. Once seated, participants were assigned one of four versions of the questionnaire, representing the four experimental between-subjects conditions. After being read the instructions by the experimenter, participants were asked to begin.

The first section measured each participant’s identification with Australia using a 14-item social identification scale with a 7-point Likert format [17]. This was added to determine whether participants identified as Australian. Furthermore, this scale served to increase the salience of an Australian social identity. The Australian social identity scale was employed as a covariate in evaluating the effects of threat level and social identity prime.

In the second section, participants were presented with one of two vignettes that described the people of Australia. Participants were told that the vignette was quoted from “Becoming an Australian Citizen©”, an official Government booklet. These vignettes implied that the information presented summarized the attitudes towards immigration held by Australians. In reality, these vignettes were developed based on the principles and language used by the CIIM and served as the social identity manipulation. For example, the vignette that promoted a one group representation included statements such as “The citizens of Australian share a common set of values that make us who we are” and “Australians are very accepting of others and no matter what race or what background one comes from, collectively we are all known as Australians”. Conversely, the vignette that promoted a separate-groups representation included statements such as “It is difficult to say that people from different races and backgrounds can be collectively known as one group, Australian” and “Citizens of Australia do not share a common set of values, we are a nation made up of separate people from separate groups”. These vignettes were refined through pilot testing, which required three samples of 10–15 each and two refinements of the “separate groups” vignette.

As a manipulation check, participants were asked to write a brief description and to answer two questions regarding the vignette they had just read. Next, participants were asked to indicate their opinion of the people of Australia. This was done by asking participants to answer two questions on a continuous 0–100 scale that assessed Australia as a group. Using a horizontal line with anchors that said “Separate groups” and “One common group”, the first question asked participants to rate the unity of the Australian people. The second question asked participants to rate the values of the Australian people on a horizontal line with anchors that said “Not Shared” and “Shared”.

The third section asked participants to answer items that described hypothetical potential marginal members (targets). The targets were presented as immigrants who had obtained Australian citizenship. To ensure that participants understood that the targets were Australian citizens of foreign descent, they were asked whether the targets were Australian citizens by birth or Australian citizens born abroad. The target descriptions were either highly threatening or non-threatening and were developed in line with the symbolic and realistic threats described in the ITT. As noted above, the six threat topics concerned the environment, language, welfare money, dogma, violence, and health. Each target’s threat pertained to just one topic. Of these, dogma and language represented symbolic threats whilst violence, welfare money, environment and health represented realistic threats. Responses from pilot tests (*N* = 25) revealed that these items elicited the predicted responses when presented as highly threatening or not at all threatening.

Targets also were described as either Canadian (ethnic ingroup), Syrian (ethnic outgroup) or without any ethnic characterization (control). The questionnaire thus described 18 targets, all of whom were male. The target descriptions were ordered randomly, and were counterbalanced by reversing the order in half of the questionnaires.

Participants were asked to judge the degree to which they viewed each target as Australian and non-Australian. A 0–20 scale was used instead of a range that might suggest proportions or percentages (e.g., 0–100), so that participants would not be given an implicit zero-sum prime. For example:

Abbas is originally from Syria. Before coming to Australia, he had contracted hepatitis B. He has on occasions visited the doctor due to complaints brought on by the recurring symptoms. He is embarrassed about having the disease and does not tell anyone about it, including potential sexual partners.

1. In your view, to what degree is Abbas Australian?
  _____ (0 to 20).
2. In your view, to what degree is Abbas Syrian?
  _____ (0 to 20).

This example is a high-threat target. The corresponding low-threat target was as follows.

Abbas is originally from Syria. He has had no prior health concerns or major illnesses. He exercises regularly and is relatively fit. He has private healthcare just in case any health problems arise in the future.

After completing the judgement task, participants were asked to give personal information such as age, sex and nationality. The experiment took approximately 20 minutes and after completion the participants were fully debriefed.

### Results

#### Analytical Methods and Issues

The Australian Identification Scale was internally consistent (Cronbach’s *α* = .92). This scale yielded a mean of 4.74 (*SD* = 0.91), significantly above the scale midpoint of 4 (95% confidence interval was [4.59, 4.90]).

To determine the success of the manipulations, two univariate analyses of variances (ANOVAs) were conducted with the type of prime as the predictor variable and the response on the continuous manipulation checks (out of 100) as the dependent variable. For the first manipulation check described above, there was a significant main effect of prime, *F* (1,127) = 8.54, *p* < .01, η^2^ = .06, CI_95_ *=* [.007, .16]. Participants were more likely to rate Australians as part of a common group when given an inclusive prime (*M* = 48.43, *SE* = 2.56) than when given an exclusive prime (*M* = 37.67, *SE* = 2.64). However, although these difference were significant and were in the predicted pattern, the mean in the inclusive prime condition was not significantly over the mid-point of 50, CI_95_ *=* [42.97, 53.94], suggesting that participants in the inclusive prime condition still rated Australians as belonging to separate groups.

For the second rating manipulation check, there was a significant main effect of prime, *F* (1,127) = 6.78, *p* = .01, η^2^ = .05, CI_95_ *=* [.003, .140]. Participants were more likely to rate the values of Australian as shared when given an inclusive prime (*M* = 64.72, *SE* = 2.15) than when when given an exclusive prime, (*M* = 56.68, *SE* = 2.27). However, although these difference were significant and were in the predicted pattern, the mean in the exclusive prime condition was significantly over the mid-point of 50, CI_95_ *=* [52.53, 60.84], suggesting that those in the exclusive prime condition still rated Australians as having shared values.

In judging the targets, some participants assigned identical mid-range scores of 10 out of 20 to Australian and Other membership. Although these are technically zero-sum (10 + 10 = 20), we did not consider them to be evidence of zero-sum thinking because, for some respondents, they may actually be an expression of uncertainty. We thus denote this type of response by the term “uncertain sum”. Clear zero-sum responses were, therefore, defined as those in which Australian and Other membership scores were unequal and summed to 20. There were 468 (19.86%) uncertain sum responses and 527 (23.36%) clear zero-sum responses. Hypotheses regarding the occurrence of uncertain sum and clear zero-sum responses were tested using mixed binary logistic regression models. These models were estimated in Stata 12 and the lme4 package [18] in R2.15, with virtually identical results in both environments.

The hypotheses regarding the effects of salient Australian identity and target threat on the sum of Australian and Other membership were tested using mixed beta GLMs [19, 20], with the degree of membership scales linearly transformed to the unit [0,1] interval by dividing the raw scores by 20. These GLMs were estimated using the NLMIXED procedure in SAS 9.2 [21] and using MCMC methods in OpenBUGS 3.1.0 [22]. Both approaches yielded very similar parameter estimates and standard errors, and the NLMIXED results are reported here.

Beta GLMs are appropriate for the degree of membership variable because it is doubly-bounded in the same sense that a probability or proportion is, whereas normal-theory regression clearly is not appropriate (see [19] for the rationale behind this). Beta GLMs have two submodels. The *location submodel* models the mean response, as in a typical regression or GLM, and uses the logit link function (as in logistic regression). The *precision submodel* models a precision parameter that influences dispersion in addition to the mean (the higher precision, the less dispersion), and uses a log link function (for more details about these submodels, see [19]).

#### Uncertain and Clear Zero-Sum Responses

Turning first to the uncertain sums responses, participants had 1.675 times greater odds of giving uncertain sums when presented with the “separate-groups” Australian identity prime than the “common” prime (*p* = .019). There was a negative interaction effect with the covariate Australian social identification (*p* = .008), such that higher scores on the covariate reduced the effect of the identity prime. For participants scoring 1 standard deviation below the mean of this covariate, the aforementioned odds-ratio increased to 3.009, whereas for those scoring 1 standard deviation above the mean this ratio reduced to 0.932. Threat level had no main effect (*p* = .301) and nor did ethnicity (*p* = .421). Topics, on the other hand, did have effects on the likelihood of uncertain sum responses. The higher-odds topics regardless of threat level were violence, welfare, and health (*p* < .0001, *p* = .039, and *p* < .0001 respectively), while the lower-odds topics regardless of threat level were language and dogma (*p* < .0001 for both).

Clear zero-sum response patterns differed considerably from those for the uncertain sums and, as expected, were more in line with the hypothesized effects. The best model for the clear zero-sum response probabilities is summarized in Table 1. The Australian identity condition had no effect, but there is a clear main effect for threat, as hypothesized (*z* = 4.449, *p* < .0001); zero-sum memberships were more common for high-threat targets. The Australian social identification covariate has no main effect, but entered into an interaction effect with ethnicity such that the gap between the probability of zero-sum responses for Unspecified versus Canadian or Syrian targets increased with increasing levels of expressed Australian social identification.

**Table 1:** Mixed Logistic Regression Fixed Effects for Zero-Sum Response Probabilities.

Coeff.	Estimate	*s*.*e*.	*z*	*p*
intercept	-2.254	0.218		
threat	0.423	0.095	4.449	< .0005
Aus. Identification	-0.145	0.217	-0.666	.505
Unspecified	0.588	0.095	6.189	< .0005
Canadian	-0.611	0.111	-5.508	< .0005
Aus. Ident x Unspec.	0.307	0.095	3.249	.001
Aus. Ident. x Canad.	-0.159	0.105	-1.518	.129
threat x Unspecified	-0.230	0.094	-2.448	.014
threat x Canadian	0.298	0.114	2.624	.009
violence	-0.407	0.161	-2.537	.011
language	0.846	0.141	6.011	< .0005
dogma	0.256	0.149	1.719	.086
welfare	-0.122	0.153	-0.795	.426
health	-0.399	0.160	-2.489	.013
threat x violence	0.021	0.161	0.131	.896
threat x language	0.413	0.141	2.932	.003
threat x dogma	0.283	0.152	1.862	.063
threat x welfare	-0.110	0.154	-0.718	.473
threat x health	-0.610	0.161	-3.788	< .0005

Unspecified and Syrian targets elicited more zero-sum responses than Canadians, almost regardless of threat level, and Unspecified targets elicited more zero-sum responses than Syrians. The effect of threat is moderated by ethnicity. There is a considerably larger effect of threat level on Canadian targets than Syrian or Unspecified targets, as shown in Table 2.

**Table 2:** Odds Ratios of Zero-Sum Responses for Threat by Ethnicity.

	Threat:	
Ethnicity:	high	low
Syrian	1.460	0.718
Canadian	1.116	0.264
Unspecified	2.182	1.484

Topics also have an impact on the likelihood of zero-sum responses, and their effects are moderated by threat. The relevant odds-ratios are shown in Table 3. In Tables 2 and 3, the design variables are coded using “effects coding” (i.e., coefficients are deviations from the mean logit). Threat levels have a very large effect on the likelihood of zero-sum membership values for targets whose descriptors focus on language or dogma, with lesser effects on environment, violence, and welfare, and a modest reverse effect on health. For high-threat targets, language and dogma descriptors have by far the highest odds of zero-sum responses.

**Table 3:** Odds-Ratios for Threat by Topic.

	Threat:	
Topic:	high	low
environment	0.874	0.357
violence	1.037	0.427
language	5.378	1.010
dogma	2.617	0.638
welfare	1.210	0.648
health	0.556	0.809

#### Sums of Membership Values Model

The best location submodel for the sum of membership values was

$$\mu_{ij}=\frac{\text{exp}\left(\gamma_{ij}\right)}{1+\text{exp}\left(\gamma_{ij}\right)},$$ (1)

where *i* indexes subjects and *j* indexes targets, and

$$\gamma_{ij}=\;\left(\beta_{0}+u_{0i}\right)+\left(\beta_{1}+u_{1i}\right)x_{1ij}+\beta_{2}x_{2i}+\beta_{3}w_{2ij}+\beta_{4}w_{3ij}+\beta_{5}x_{1ij}w_{2ij}+\beta_{6}x_{1ij}w_{3ij},$$ (2)

where *x*_1ij_ denotes threat, *x*_2*i*_ denotes Australian Identification, *w*_2ij_ = 1 if the target is Unspecified and 0 if Canadian, *w*_3ij_ = 1 if the target is Canadian and 0 if Unspecified, and both are -1 if the target is Syrian. Random effects $u_{0i}\sim \;N\left(0,\sigma_{0}^{2}\right)$ and $u_{1i}\sim \;N\left(0,\sigma_{1}^{2}\right)$ were treated as independent. The location submodel parameter estimates, standard errors, t statistics, significance levels and 95% confidence intervals are shown in the upper part of Table 4.

**Table 4:** Parameter Estimates for Membership Sums Beta GLM.

						95%	CI
Effect	Param.	Estimate	*s*.*e*.	*t*	*p*	Lower	Upper
Location submodel							
intercept	*β*_0_	0.445	0.079	5.660	< .0005	0.289	0.600
threat	*Β*_1_	-0.290	0.028	-10.270	< .0005	-0.346	-0.234
Aus.Ident.	*β*_2_	0.113	0.079	1.440	.153	-0.043	0.269
Unspecified	*β*_3_	-0.017	0.020	-0.830	.409	-0.056	0.023
Canadian	*Β*_4_	0.022	0.023	0.980	.327	-0.023	0.067
threat x Unspec.	*Β*_5_	0.087	0.020	4.320	< .0005	0.047	0.126
threat x Canad.	*Β*_6_	-0.140	0.023	-6.060	< .0005	-0.186	-0.094
Precision submodel							
intercept	*δ*_0_	2.118	0.031	69.360	< .0005	2.058	2.179
Common ident.	*δ*_1_	0.083	0.030	2.750	.007	0.023	0.143
Unspecified	*δ*_2_	0.166	0.045	3.720	< .0005	0.078	0.255
Canadian	*δ*_3_	-0.204	0.043	-4.720	< .0005	-0.289	-0.118

On average, participants returned subadditive ratings (i.e., they summed to more than 20, *M* = 23.56, *SD* = 8.12), so there was a general tendency to allow targets greater combined group membership than zero-sum additivity to 20 would dictate. In the location submodel there is a main effect for threat that is moderated by ethnicity. There was no effect from the Australian identity condition, but there was an effect from target threat.

Table 5 displays the mean summed membership ratings for threat by ethnicity (on the original 0–20 scale). The main effect for threat yields higher combined group membership for low-threat targets, as hypothesized. There is no main effect for either ethnicity (*p* = .409 and *p* = .327), and the interaction effect in the location submodel is simply a greater difference across threat-levels for Canadian targets than for the other two targets.

**Table 5:** Mean Summed Membership Ratings: Threat by Ethnicity.

	Threat:	
Ethnicity:	high	low
Syrian	21.66	25.46
Canadian	20.53	27.37
Unspecified	21.58	24.88

The best precision submodel was

$$\tau_{ij}=\delta_{0}+\delta_{1}x_{2ij}+\delta_{2}w_{2ij}+\delta_{3}w_{3ij},$$ (3)

where *x*_2ij_ = 1 if the Australian identity is the common-identity version and -1 if it is the separate-identity version, and *w*_2ij_and *w*_3ij_ are the ethnicity indicator variables described above. The coefficient estimates and relevant statistics are displayed in the lower part of Table 4. There is greater precision (i.e., less dispersion and therefore greater consensus among participants) for ratings under the common identity condition, and precision is highest for the Unspecified and lowest for Canadian targets.

### Study 1 Discussion

The study reported here investigated the joint effects of perceived threat and superordinate identity on the phenomenon that has been called marginalizing racism in two novel ways: (1) Zero-sum degrees of membership in an in-group and an out-group, and (2) Summed membership in an in-group and an out-group. The hypotheses tested in this experimental setup were:

1. High threat targets yield:
  - Higher likelihood of zero-sum membership assignments (supported)
  - Lower summed membership (supported)
2. An inclusive superordinate in-group identity for low-threat targets yields:
  - Lower likelihood of zero-sum membership assignments (not supported)
  - Higher summed membership (not supported)
3. Canadian targets compared to Syrian targets yield:
  - Lower likelihood of zero-sum membership assignments (supported)
  - Higher summed membership (not supported)

Both parts of Hypothesis 1 received support, whereas neither part of Hypothesis 2 was supported. Additionally, there were moderations of the impact of threat. The effect of threat on zero-sum membership assignments was moderated both by ethnicity and target descriptor. Canadian targets had the largest threat effect, while for high-threat targets, language and dogma descriptors had the highest odds of zero-sum assignments. Likewise, the impact of threat on the sum of membership values also was largest for Canadian targets. Unspecified and Syrian targets elicited more zero-sum responses than Canadians, thereby supporting Hypothesis 3a. Contrary to Hypothesis 3b, there was no discernible impact of ethnicity on combined group membership. Finally, degree of Australian identification played only a minor role, and in particular did not moderate the effects of threat or topic, and only moderating the effect of ethnicity on the number of zero-sum responses.

## Study 2

Our findings suggest several future directions for research on this topic. Perhaps the most fundamental of these is the question of whether, if people believe that stronger membership in one group implies weaker membership in another group, the converse also holds. In other words, is belief in a zero-sum restriction on group membership symmetric? In the context of dual national membership or citizenship arising from immigration, one possibility is that people will believe that the degree of immigrants’ membership in their nation of origin determines the degree of membership in their adopted country to a stronger extent than vice-versa. That is, people will endorse more strongly the notion that greater membership of immigrants’ identification in their original nation entails lesser membership in their adopted nation, than the notion that greater membership in their adopted nation entails lesser membership in their original nation.

If this hypothesis is borne out, then marginalizing racism as manifested by zero sum beliefs regarding simultaneous group membership hinges on a framing effect: The ordering of the elements in statements of such beliefs. Zero-sum statements have the form “The more X for A, the less Y for B”, where X and Y are resources (in many cases X and Y are the same resource) and A and B are consumers. These statements have four permutations:

1. “The more X for A, the less Y for B”
2. “The less X for A, the more Y for B”
3. “The more Y for B, the less X for A”
4. “The less Y for B, the more X for A”

A strict zero-sum believer should regard these four statements as equivalent. However, when X and Y are strengths of identification with one’s original and adopted nationalities respectively (and A = B = immigrant), we hypothesize that permutations 1 and 4 will be more strongly endorsed than permutations 2 and 3. In Study 2 we tested this hypothesis on samples from four countries: China, India, the U.K., and the U.S.A.

### Method

#### Design

Study 2 was embedded in an experiment whose primary purpose was an investigation of the impact of individual differences variables and priming on endorsement of zero-sum items [23]. Study 2 employed a 4-between by 2-within design, where the first factor refers to the permutation of the zero-sum statements and the second refers to the fact that each participant rated two versions of a zero-sum statement about immigrants (see Procedures and Materials).

The primary purposes of the study in which Study 2 was embedded were twofold. The first purpose was to investigate the effects of the four permutations on endorsement levels. Eight zero-sum statements were selected for this, based on two previous studies of these effects. A quantum probabilistic explication has been developed in [24], of the three endorsement patterns identified with these statements that were replicated from earlier studies and that were consistent across samples.

The second purpose was to ascertain whether scores on two psychopathy inventories were associated with zero-sum statement endorsement levels. The inventories were the LSRP [25] and the SRP II [26]. This was motivated by the findings of an earlier investigation of the relationships between zero-sum beliefs and individual differences variables that included scales measuring the big-5 personality factors, social dominance orientation, and competitive world view. Both studies are reported in [23].

Thus, the current study had a 4-by-2-between by 8-within design. The additional between-subjects manipulation counterbalanced the order in which participants received the zero-sum statements and the bank of items from the psychopathy scales. An order effect is reported in [23] whereby receiving the psychopathy items first lowered the endorsement of zero-sum statements. This was a main effect only and therefore did not moderate any of the results reported here.

Prior to this paper, none of the data in [23, 24] have been published. Two of the eight zero-sum statements are analysed in this paper because they were designed to tap into participants’ beliefs and attitudes regarding immigrants’ identities in the country to which they have immigrated. The remaining six statements pertain to topics that are unrelated to group membership or group identification.

#### Participants

After the study was granted approval from The ANU Human Research Ethics Committee (protocol 2012/430), participants were recruited by Qualtrics from China, India, the U.K., and the U.S.A. Participant data were excluded from the analyses if the participant spent less than 5 minutes on the survey, gave inconsistent answers to questions, or did not answer relevant questions. After these exclusions, Chinese participants numbered 496 with 50.4% female and mean age of 30.3 years (*SD* = 7.8); Indian participants numbered 503 with 49.9% female and mean age of 34.4 years (*SD* = 14.2); U.K. participants numbered 494 with 49.8% female and mean age of 49.6 years (*SD* = 14.4); and U.S.A. participants numbered 498 with 49.8% female and mean age of 43.8 years (*SD* = 15.6).

#### Procedure and Materials

The study was conducted online, using the Qualtrics survey facility. First, participants were presented with an information screen describing the study, and given the option to exit the survey or consent to participate by clicking on a “Continue” button, which they had been informed would constitute granting consent (They could still opt out of the study at any time. This procedure was approved by the ANU Human Research Ethics Committee, protocol 2012/430). Participants then completed three scales measuring psychopathy, cold-heartedness, and interpersonal arrogance (as part of the study reported in [23, 24]), and rated eight zero-sum statements. The order of the scales and zero-sum statements was counter-balanced.

Examples of the two items relevant to this study are shown below as presented (in English) to the U.S.A., U.K., and India samples. Both examples are in the form hypothesized to receive the strongest endorsement of the four permutations.

1. Consider Hama Al-Bayati, who immigrated to the U.S.A. (U.K., India) 5 years ago from Iraq. The more “Iraqi” he is, the less “American” (“British”, “Indian”) he will be.
2. Consider Ali Al-Husseni, who immigrated to Germany 5 years ago from Iraq. The more “Iraqi” he is, the less “German” he will be.

The first statement clearly is intended to tap into participants’ attitudes about immigrants’ identity in their own country. The second statement is intended for comparing participants’ attitudes about dual national identity when the target has migrated to a country other than their own.

The Chinese survey was presented in simplified Chinese, having been translated into Chinese and back-translated into English by two native-speakers of Chinese who are fluent in English. The immigration items are not relevant in Chinese society, so substitutes were constructed that describe a Chinese citizen immigrating from one province to another. The substitute for statement 1 had the person immigrating to the home province of the respondent, while the substitute for statement 2 had the person immigrating to another province (Xinjiang).

Participants were asked to indicate on a seven-point Likert scale the extent to which they agreed or disagreed with their version of both statements. Participants in the first condition received permutation 4 of the Hama Al-Bayati question and permutation 1 of the Ali Al-Husseni question; those in the second condition received permutation 2 of the Hama Al-Bayati question and permutation 3 of the Ali Al-Husseni question; those in the third condition received permutation 3 of the Hama Al-Bayati question and permutation 2 of the Ali Al-Husseni question; and those in the fourth condition received permutation 1 of the Hama Al-Bayati question and permutation 4 of the Ali Al-Husseni question.

Responses were analyzed using ordinal logistic regression with the VGAM package [27] in R. The two questions were analyzed in separate models. We included national identity in the models but ignored the experimental variables not relevant to this study (i.e., whether participants completed the zero-sum statements first or not, and the forms of the other zero-sum statements).

### Results and Discussion

#### Hama Al-Bayati question

**Fig 1:**
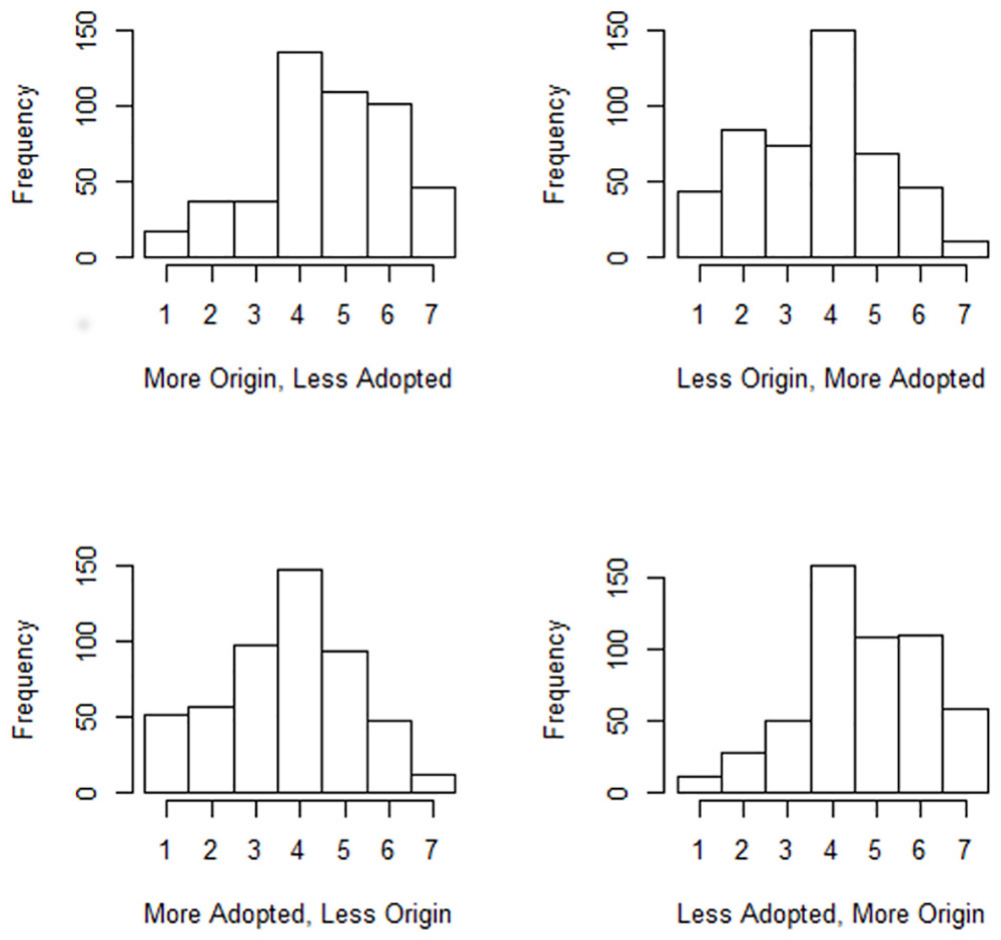
Histograms for the Hama Al-Bayati question.

The histograms in Fig 1 indicate that, as hypothesized, the “More Origin, Less Adopted” (version 1) and “Less Adopted, More Origin” (version 4) versions of the Hama Al-Bayati question are more strongly endorsed than the other two versions. The relevant coefficients from the ordinal logistic regression model are reported in Table 6. The model uses version 4 of the zero-sum statements and the U.S.A. as the base-groups, so all coefficients reflect differences from those two “benchmarks”.

**Table 6:** Ordinal Regression Coefficients for the Hama Al-Bayati Question.

Coeff.	Estimate	*s*.*e*.	*z*	*p*
U.K.	1.44	0.24	6.11	< .0005
India	0.44	0.22	1.94	.026
China	1.01	0.23	4.43	< .0005
V1*	0.08	0.22	0.37	.356
V2	-0.71	0.23	-3.13	.001
V3	-0.83	0.23	-3.67	< .0005
U.K.: V1	-0.17	0.32	-0.51	.305
U.K.: V2	-1.79	0.33	-5.42	< .0005
U.K.: V3	-1.59	0.32	-4.96	< .0005
India: V1	0.60	0.31	1.91	.028
India: V2	-0.19	0.32	-0.58	.281
India: V3	0.70	0.32	2.19	.014
China: V1	-0.42	0.32	-1.33	.092
China: V2	-0.20	0.32	-0.61	.271
China: V3	-0.16	0.32	-0.50	.309

*V = version, e.g., V1 = version 1

The main effects pattern (i.e., for the U.S.A. sample) for versions 1–3 support the hypothesis; endorsement of version 1 does not differ significantly from endorsement of version 4, whereas versions 2 and 3 are less strongly endorsed than version 4. The U.K. sample has a stronger pattern than the U.S.A. in support of this hypothesis, and the Chinese pattern of endorsement does not differ significantly from the U.S.A. pattern. The sole finding contrary to the hypothesis is the India: The V3 effect, which results in no significant difference between endorsement of versions 3 and 4 in the Indian sample.

#### Ali Al-Husseni question

**Fig 2:**
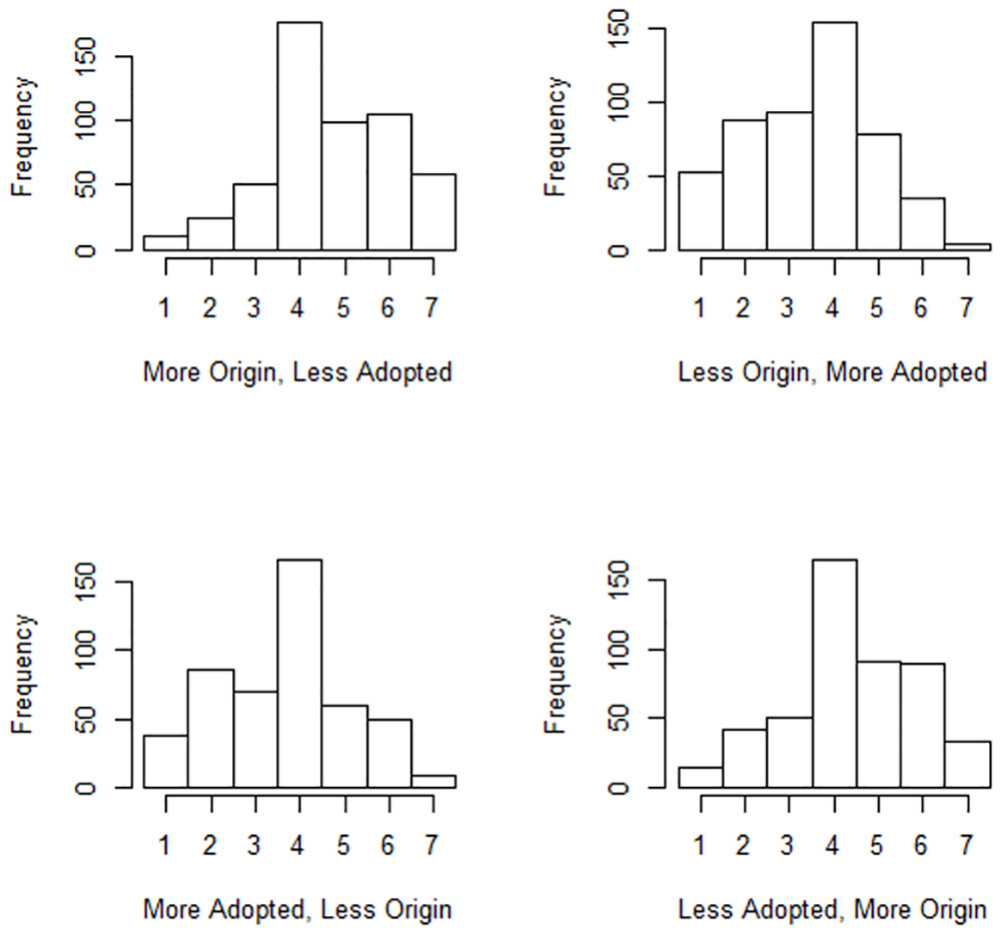
Histograms for the Ali Al-Husseni question.

The histograms in Fig 2 indicate that, as hypothesized, the “More Origin, Less Adopted” (version 1) and “Less Adopted, More Origin” (version 4) versions of the Ali Al-Husseni question are more strongly endorsed than the other two versions. The relevant coefficients from the ordinal logistic regression model are reported in Table 7. As before, the model uses version 4 of the zero-sum statements and the U.S.A. as the base-groups, so all coefficients reflect differences from those two “benchmarks”.

**Table 7:** Ordinal Regression Coefficients for the Ali Al-Husseni Question.

Coeff.	Estimate	*s*.*e*.	*z*	*p*
U.K.	1.07	0.24	4.56	< .0005
India	0.54	0.22	2.38	.009
China	0.49	0.23	2.15	.016
V1*	0.19	0.23	0.86	.195
V2	-0.57	0.23	-2.47	.007
V3	-0.88	0.23	-3.88	< .0005
U.K.: V1	0.16	0.32	0.51	.306
U.K.: V2	-1.44	0.33	-4.34	< .0005
U.K.: V3	-1.08	0.32	-3.36	< .0005
India: V1	0.43	0.31	1.38	.084
India: V2	-0.36	0.32	-1.13	.128
India: V3	-0.02	0.32	-0.07	.471
China: V1	-0.03	0.32	-0.10	.461
China: V2	0.17	0.32	0.52	.303
China: V3	0.06	0.32	0.17	.431

*V = version, e.g., V1 = version 1

As before, the main effects pattern (i.e., for the U.S.A. sample) for versions 1–3 support the hypothesis; endorsement of version 1 does not differ significantly from endorsement of version 4, whereas versions 2 and 3 are less strongly endorsed than version 4. Again, the U.K. sample has a stronger pattern than the U.S.A. in support of this hypothesis. This time, neither the Chinese nor the Indian patterns of endorsement differ significantly from the U.S.A. pattern.

#### Study 2 Discussion

The findings suggest an extension of the concept of marginalizing racism to incorporate a type of reasoning which holds that stronger degree of membership in or identification with one’s original nationality constrains membership in a new nationality to a greater extent than stronger membership in the new nationality constrains membership in the original nationality. Notably, this pattern generally held across the four nations regardless of whether the adopted country (province) was their own or Germany (Xinjiang). The robustness of this pattern indicates that it generalizes across cultures and is relatively independent of the target country (province).

## General Discussion

Whether memberships in multiple groups are treated as zero-sum depends on contextual factors. Study 1 demonstrated this claim by showing effects from threat and ethnicity on the likelihood of zero-sum national identity membership assignments. Negative aspects such as perceived threat and negative ethnic stereotypes may be fairly obvious candidates for triggering and bolstering zero-sum beliefs about multiple group membership, but some findings in Study 1 point to joint effects that merit further investigation and theoretical development. Chief among these is the apparently greater impact of perceived threat on membership attributions to Canadian than to Syrian or unspecified targets. It seems plausible that, because of the cultural similarities between Australians and Canadians, the Australian participants in Study 1 found that the threat descriptors were more strongly counter-normative for Canadian targets than the other two.

Speculation regarding the motives behind zero-sum beliefs about national identity suggests an avenue of investigation into when in-group membership becomes viewed as zero-sum for “native” in-group members. The ingroup projection model (IPM) provides some useful concepts for this purpose. According to this model, ingroup-outgroup comparisons are made via judgments of the relative prototypicality of the groups in a relevant superordinate category [28]. Positively distinctive aspects of the ingroup are “projected” onto this superordinate category, so that the ingroup appears more prototypical than the outgroup in such comparisons.

Wenzel et al. point out that the IPM and CIIM make opposite predictions regarding the consequences of ingroup members who also identify with the superordinate category. They resolve this disagreement by speculating that when ingroup projection occurs, if dual identity yields perceived intergroup similarity it leads to more positive intergroup attitudes. In contrast, if it yields intergroup differentiation with reference to a superordinate prototype it leads to more negative intergroup attitudes [28].

This account of the IPM and CIIM suggests two ways in which zero-sum beliefs about ingroup and outgroup membership may arise. First, the outgroup may be perceived as so dissimilar to the ingroup that they are genuinely non-overlapping categories, so that the outgroup is not seen to be a member of the superordinate category, and therefore membership in them is zero-sum.

Second, zero-sum membership may arise if intergroup differentiation is sufficiently competitive that prototypicality in the superordinate category becomes zero-sum. Obvious dimensions that would promote this development are those on which the groups are perceived to be in competition with one another (for nations, examples include sporting prowess, military power, or influence over other countries).

The findings in Study 2 raise both theoretical and methodological issues. The methodological issue raised by Study 2 pertains to the measurement of zero-sum beliefs. Clearly, the four permutations of zero-sum propositions are unequally endorsed, and there appears to be a consistent pattern in the relative strengths of endorsement. In short, the self-reported strength of “zero-sum” beliefs depends on how propositions about those beliefs are presented to respondents, thereby complicating the measurement of zero-sum thinking. This topic is systematically investigated in [24], but we provide a brief summary of their findings here.

Two heuristics are isolated in [24] regarding perceptions about resource consumers that appear to influence endorsement of zero-sum propositions. First, there is an *asymmetric potency* effect, whereby in some propositions one of the resource consumers (A, say) is perceived as more powerful or potent than the other (B). In that case, propositions of the form “The more X for A, the less X for B” and “The less X for A, the more X for B” are more strongly endorsed than the other two versions. An example from [24] exhibiting this pattern is “Devoting more (less) time to work takes time away from (makes more time for) personal relationships”. Second, there is an *asymmetric gains potency* effect, whereby A’s acquiring more of X is seen as more potent than B’s acquisition *and* than A acquiring less of X. In this case, people infer that the more A receives the less there is for B, and the less B receives the more there is for A. An example from [24] exhibiting this pattern is the stronger endorsement of “When the rich get richer the poor get poorer” and “When the poor get poorer the rich get richer” than the remaining two permutations of this statement. Thus, the pattern found in Study 2 for both questions was the *asymmetric gains potency* effect.

The primary theoretical issue involves inferences that people make about multiple group membership when a transition is made from membership in one group to membership in another. The effect of this transition is at the heart of many expressions of marginalizing racism. A simple intuition has it that a residue of one’s original group identity remains even after one has severed ties with that group and joined another. This appears to explain the experiences of Turkish immigrants in Germany.

Study 2 suggests a more subtle version of this “original sin” account. The findings indicate a tendency for people to believe that stronger membership in the original group entails weaker membership in the new group, and vice-versa. However, they give less endorsement to the idea that *weaker* membership in the original group entails *stronger* membership in the new group, or vice-versa. The flow of membership, so to speak, from one group to another is one-way. Strengthening one’s original group membership leaches membership from the newly adopted group, but neither strengthening membership in the adopted group nor weakening original group membership has much effect on membership in the other group. An obvious extension of this line of inquiry is to ascertain whether this pattern holds regardless of how favourably either the original or adopted group is regarded, or any history of conflict between the groups.

Neither study linked zero-sum beliefs about multiple group membership with zero-sum beliefs about inter-group relations. While there have been investigations into zero-sum beliefs about group competition [29–31], these have not linked such beliefs with attitudes or beliefs regarding multiple group membership. This would seem an obvious next step to take. Study 2 included a zero-sum statement that was permutations on “If the rate of immigration is increased then there will be fewer jobs to go around”. Despite the fact that this statement is not specific about ethnic identities, correlations between responses to it and to the Hama Al-Bayati and the Ali Al-Husseni questions were positive for all four national samples (.216, .202, .225, and .142 for the Hama Al-Bayati and .260, .258, .231, and .219 for the Ali Al-Husseni, in the USA, UK, India, and China samples respectively). This circumstantial evidence motivates an investigation into the relationship between zero-sum beliefs about group membership and/or identity, and zero-sum beliefs about the corresponding inter-group consumption of resources.

We began this paper by suggesting that membership may not be a simple dichotomy but may be a matter of degree, and that the process of marginalizing racism could be investigated by allowing people to express degrees in their judgments about group membership. This suggestion has paid off, by sharpening our understanding of marginalizing racism in two respects. First, we recognized that marginalizing racism can be expressed as a belief that ingroup and outgroup membership is zero-sum. This recognition allowed us to explore, in Study 1, the manner in which threat and inter-group relations combine to modulate this process of zero-sum thinking. Second, we also recognized that people may believe membership can be zero-sum in transfers from one group to another but not the reverse, as demonstrated in Study 2 with replication across four nations. We have, thus, shown that marginalizing racism is more than a belief that people retain a “stain” from membership in their original group (e.g., as a form of essentialist thinking; see e.g.,[32]). Marginalizing racism also manifests itself in the form of strict zero-sum and conditional zero-sum beliefs about multiple group memberships.
